# Comparison of Multi-Tensor Diffusion Models' Performance for White Matter Integrity Estimation in Chronic Stroke

**DOI:** 10.3389/fnins.2018.00247

**Published:** 2018-04-23

**Authors:** Olena G. Filatova, Lucas J. van Vliet, Alfred C. Schouten, Gert Kwakkel, Frans C. T. van der Helm, Frans M. Vos

**Affiliations:** ^1^Department of Biomechanical Engineering, Delft University of Technology, Delft, Netherlands; ^2^Quantitative Imaging Group, Department of Imaging Physics, Delft University of Technology, Delft, Netherlands; ^3^Laboratory for Biomechanical Engineering, Institute for Biomedical Technology and Technical Medicine, University of Twente, Enschede, Netherlands; ^4^Department of Rehabilitation Medicine, MOVE Research Institute Amsterdam, Amsterdam Neurosciences, VU University Medical Center, Amsterdam, Netherlands; ^5^Department of Radiology, Academic Medical Center, Amsterdam, Netherlands

**Keywords:** diffusion MRI, brain, stroke, diffusion tensor imaging/methods, rehabilitation outcomes, motor performance, anatomic lateralization

## Abstract

Better insight into white matter (WM) alterations after stroke onset could help to understand the underlying recovery mechanisms and improve future interventions. MR diffusion imaging enables to assess such changes. Our goal was to investigate the relation of WM diffusion characteristics derived from diffusion models of increasing complexity with the motor function of the upper limb. Moreover, we aimed to evaluate the variation of such characteristics across different WM structures of chronic stroke patients in comparison to healthy subjects. Subjects were scanned with a two b-value diffusion-weighted MRI protocol to exploit multiple diffusion models: single tensor, single tensor with isotropic compartment, bi-tensor model, bi-tensor with isotropic compartment. From each model we derived the mean tract fractional anisotropy (FA), mean (MD), radial (RD) and axial (AD) diffusivities outside the lesion site based on a WM tracts atlas. Asymmetry of these measures was correlated with the Fugl-Meyer upper extremity assessment (FMA) score and compared between patient and control groups. Eighteen chronic stroke patients and eight age-matched healthy individuals participated in the study. Significant correlation of the outcome measures with the clinical scores of stroke recovery was found. The lowest correlation of the corticospinal tract *FA*_asymmetry_ and FMA was with the single tensor model (*r* = −0.3, *p* = 0.2) whereas the other models reported results in the range of *r* = −0.79 ÷ −0.81 and *p* = 4E-5 ÷ 8E-5. The corticospinal tract and superior longitudinal fasciculus showed most alterations in our patient group relative to controls. Multiple compartment models yielded superior correlation of the diffusion measures and FMA compared to the single tensor model.

## Introduction

Unilateral loss of motor function is a frequent consequence of stroke. White matter changes in the corticospinal tract (CST) and the posterior limb of the internal capsule (PLIC) are known to correlate to motor impairment in stroke patients (Cho et al., 2007; Schaechter et al., 2008; Jang et al., 2010; Park et al., 2013; Song et al., 2014). Therefore, accurate measurements of white matter changes could be an indicator of stroke severity.

A commonly used technique to assess white matter integrity is diffusion Magnetic Resonance Imaging (dMRI). Specifically, it measures the ability of water molecules to move freely in the surrounding tissue. Importantly, normal white matter (WM) shows high diffusivity along axons and low across axons, whereas gray matter (GM) shows more isotropic diffusion patterns (Neil, 2008). The diffusion is conventionally modeled by a mathematical construct called a tensor, which can be visualized by an ellipsoid. It represents the shape of the local diffusion, from which measures are derived such as the mean diffusivity (MD) and the fractional anisotropy (FA). FA is a measure of anisotropy or pointedness of a diffusion ellipsoid and MD represents the mean diffusion in a voxel.

dMRI has proven to be a versatile tool and used in a wide range of applications. Particularly, FA_asymmetry_ was utilized by Byblow et al. (2015) who aimed to predict which patients will have spontaneous recovery. In a severe patient category without transcranial magnetic stimulation (TMS) evoked potentials, patients with initially small FA_asymmetry_ would still show some recovery, in contrast to patients with a large FA_asymmetry_. For the purpose of building a linear regression model, FA_asymmetry_ was binarized (< >0.15) and became the only significant predictor of change in the FMA score. Therefore, FA_asymmetry_ could be a good predictor of the stroke severity and potential recovery.

Several studies with animal models of stroke found that structural white matter changes in both the ipsi- and contralesional hemispheres play an important role in motor recovery (Dancause et al., 2005; Brus-Ramer et al., 2007). Furthermore, many studies have employed measures of brain asymmetry to study stroke outcome (Bhagat et al., 2008; Lindenberg et al., 2010; Puig et al., 2010; Park et al., 2013; Cunningham et al., 2015). For example, the asymmetry of FA along the PLIC in the ipsi- vs. contralesional hemisphere had higher correlation to upper limb motor functioning than functional MRI responses of cortical motor areas in chronic stroke patients (3–9 months post stroke) (Qiu et al., 2011). Ratios of FA between the affected and unaffected hemispheres were also used to study neuronal alterations immediately after stroke in hyper-acute ischemic stroke patients (Bhagat et al., 2008) and longitudinally from 1 week to 1 year (Yu et al., 2009). In the hyper-acute phase, the fractional anisotropy in the lesion relative to the healthy side was not consistent among patients, but elevated in some of them. After the first 24 h this FA ratio showed more consistent reductions (Bhagat et al., 2008). Subsequently, after 3 months, changes in the anisotropy ratio stabilized (Yu et al., 2009). Still, the exact patterns of change in diffusion properties of WM after stroke remain unclear.

Although white matter alterations are well-known to correlate with motor functioning in stroke patients, the exact etiology of these changes remains unknown. It has been observed, for example, in Buma et al. (2013) that after stroke a general white matter deterioration takes place. However, as described above, most previous studies have merely focused on the CST and PLIC. Furthermore, a single diffusion ellipsoid was classically used to model the local diffusion of the water molecules. This conventional model is known to be inadequate for the characterization of diffusion in complex structures such as crossing fibers found, for example, at the intersection of the CST and corpus callosum. Importantly, (Jeurissen et al., 2013) showed that crossing fibers are present in 60-90% of the diffusion data. The benefit of more sophisticated diffusion modeling has already been reported for other applications (Caan et al., 2010; Arkesteijn et al., 2015; Yang et al., 2015) and in a recent work of Archer et al. (2017), where a two-compartment model representing free water and white matter tissue is used to study a relation between FA_asymmetry_ and grip strength in chronic stroke subjects.

A single-tensor model is often applied to analyze dMRI in both chronic and longitudinal stroke studies (Lindenberg et al., 2012; Ma et al., 2014). A systematic review was presented by Kumar et al. (2016). We hypothesize that more complex diffusion models are better equipped to detect subtle WM changes after stroke. This hypothesis also underlies the use of a two-compartment model in Archer et al. (2017), which adapted an approach by Pasternak et al. (2009) to model a tissue and a free water compartment.

Multi-tensor models are a logical extension of the traditional single tensor model. Certainly, other higher order models exist, such as spherical deconvolution (SD) approaches. Instead of assuming a specific number of fibers, these models assume a distribution of fiber orientations. This allows to express the diffusion weighted signal as the spherical convolution of the fiber orientation distribution function and a response function representing the signal of a single fiber population (Tournier et al., 2004). However, a response function is unknown and generally assumed to be the same for the whole brain possibly leading to spurious results (Parker et al., 2013).

This paper aims to evaluate four diffusion models of increasing complexity based on the strength of the relation between the estimated WM properties and the clinical outcomes of chronic stroke patients. One of the motivating factors for the choice of these models is to validate their suitability for stroke patients and the feasibility of a model comparison per model parameter. The outcome parameters of the diffusion models are physically meaningful and directly comparable to each other. Moreover, the asymmetry between the healthy and afflicted hemispheres in the patient group is compared with the asymmetry in healthy controls in order to assess which WM properties are most relevant for such a comparison. We start with an application of the traditional single tensor model and build up to a so-called bi-tensor model with isotropic compartment in order to identify the smallest differences in diffusivities. This bi-tensor model especially takes into account that the diffusion may be affected by free water due to a cerebrovascular accident. We hypothesize that there are significant differences in diffusivity measures in several tracts between the two hemispheres. Furthermore, we anticipate that the application of sophisticated models allows a more sensitive analysis than the conventional approach.

## Methods

### Cohort

Subjects were included after informed consent and with permission of the Medical Ethics Committee of the Vrije Universiteit Medical Center, Amsterdam. The trial protocol was registered on 23 October 2013 at the Netherlands Trial Register (identifier NTR4221). Inclusion criteria for the subjects suffering from chronic stroke were: upper limb paresis, ability to sit without support (National Institutes of Health Stroke Scale item 5a/b > 0), age > 18, first-ever ischemic hemispheric stroke, >6 months post stroke. Exclusion criteria were: previously existing pathological neurological conditions or orthopedic limitations of the upper limb that would affect the results, botuline-toxine injections, or medication that may have influenced upper limb function in the past 3 months, general MRI contra indications (claustrophobia, pacemaker, or other metallic implants), high risk of epilepsy.

Patients (*n* = 18) were consecutively included from April 22, 2015 to February 29, 2016. Additionally, 8 controls with matching mean age were recruited for comparison in the period between April 9, 2015 and November 22, 2016. Post-hoc sample size analysis for this unmatched case-control study at the two-sided significance level of 0.05 indicated the power (chance of detecting) of 90% assuming a proportion of 44% of controls and an effect size of 0.5 (Kelsey et al., 1996; Charan and Biswas, 2013). Based on this analysis we stopped inclusion as we reached the mentioned number of patients and controls.

The patient population consisted of 18 first-ever ischemic stroke patients (12 men); median age: 60 (IQR: 51–67); 8/18 patients had an impaired left hand; for 9/18 patients the dominant hand was impaired; median FMA score: 57 (IQR: 24.75–61.5); median Action Research Arm Test (ARAT) score: 54 (IQR: 16.25–56.75). The control group consisted of 8 healthy subjects (7 men); median age: 59.5 (IQR: 55.25–62.25).

### MRI protocol

Image acquisition was performed with a 3T MRI scanner (Discovery MR750, GE Medical Systems). The diffusion-weighted MRI (dMRI) acquisition protocol involved 40 non-collinear gradient directions uniformly sampled over a sphere for each of two b-values: 1,000 and 2,000 s/mm^2^; *TE* = 100 ms, *TR* = 7, 200 ms, field of view FOV = 240 × 240 mm^2^, imaging matrix = 96 × 64 (zero padded to 256 × 256), 52 consecutive slices with a thickness of 2.5 mm, acquisition time 12.5 min. This allowed for whole brain coverage. Data for each b-value were acquired as separate scans together with five non-diffusion weighted images (i.e., per b-value).

### Data processing

**Figure 1:**
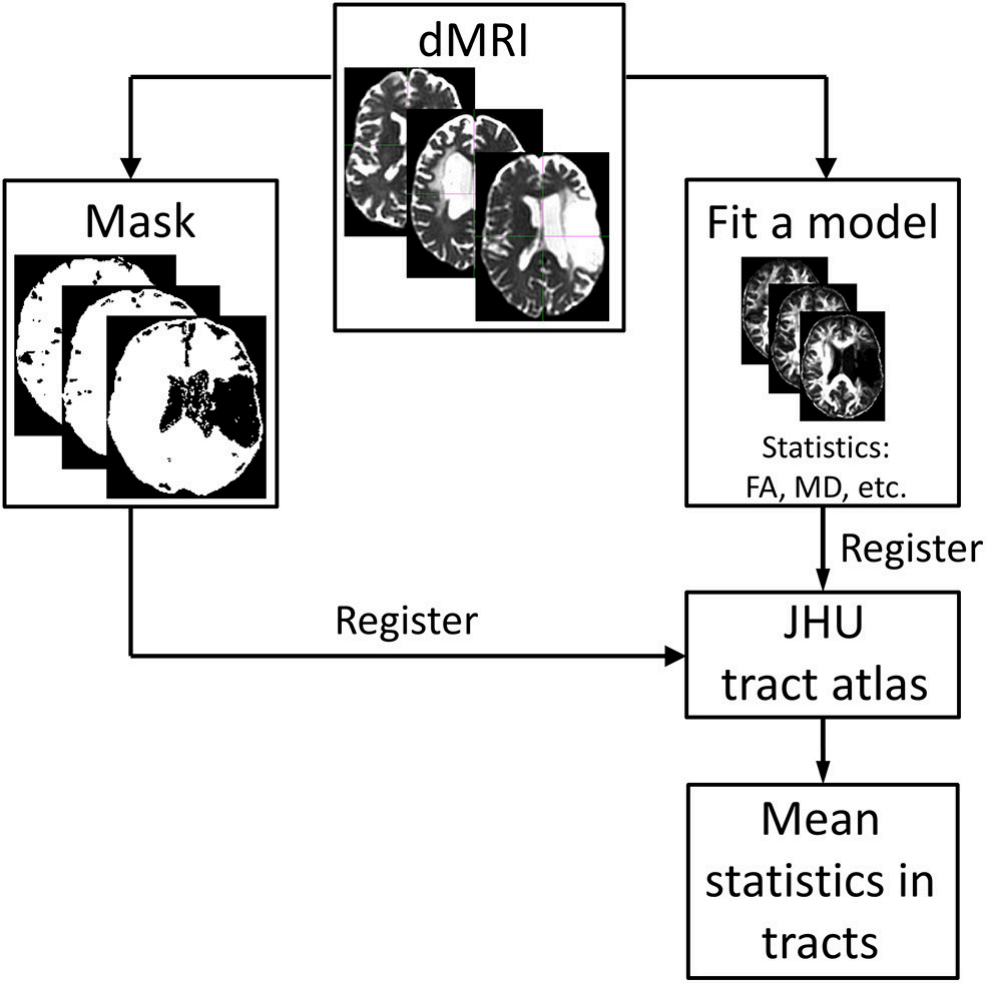
Overview of the analysis steps starting with the pre-processed dMR images. A diffusion tensor model is fitted to the data (four different diffusion models were used in this study). Masks excluding the subject-specific lesion site are created based on the thresholded isotropic compartment of the bi-tensor model with isotropic compartment. The results of this step are registered to the white-matter tract atlas (https://neurovault.org/media/images/264/JHU-ICBM-tracts-maxprob-thr25-1mm.nii.gz) and mean values of the outcome parameters are calculated for each tract.

Figure 1 presents an overview of the analysis steps described below.

#### Pre-processing

dMRI data were preprocessed using FSL v5.0 (http://fsl.fmrib.ox.ac.uk/fsl/; Jenkinson et al., 2012). The acquired DWIs were corrected for motion and eddy current distortion by affine coregistration to the reference *b*_0_-image (using FSL eddy_correct). Gradient directions were reoriented according to the rotation component of the affine transformation. Datasets of the same subject with b = 1,000 and b = 2,000 s/mm^2^ different diffusion weighting were coregistered with each other using FSL flirt affine registration with six degrees of freedom.

#### Diffusion models

The following four diffusion models of increasing complexity were fitted to the diffusion data of each voxel using the so-called maximum likelihood estimation as described, for example, in Caan et al. (2010):

1. Single-tensor model: A single tensor. The tensor shape is unconstrained. Images were generated representing FA, MD, axial diffusivity (AD), and radial diffusivity (RD). Axial diffusivity is the principal eigenvalue of the diffusion tensor and is often considered to represent the diffusion along a fiber tract. Radial diffusivity equals the mean of the secondary eigenvalues and is often taken to represent the diffusivity perpendicular to a tract.
2. Single tensor with an isotropic compartment: A single tensor compartment accompanied by an isotropic, free water compartment. The shape of the tensor is unconstrained. Unlike in Arkesteijn et al. (2015), not even a trace constraint is applied. This model yielded the same parameters as the single tensor model derived from the fiber compartment, and in addition a volume fraction characterizing the amount of free water in all voxels.
3. Bi-tensor model (Caan et al., 2010): A model consisting of two tensor compartments with the same shape (symmetric tensors with equal AD and RD, and thus same FA), but variable volume fractions (i.e., relative contribution to a voxel) and arbitrary orientations. This model represented two white matter bundles simultaneously present in a voxel. It yielded the same parameters as the single tensor model due to the presumed identical shape of the two tensors. The volume fraction was not considered for further analysis as it is affected by (arbitrary) partial volume effects.
4. Bi-tensor model with an isotropic compartment (Yang et al., 2016): A model combining the representation of crossing nerve bundles and free water diffusion in a single voxel. The two estimated tensors are restricted to have the same shape as in the bi-tensor model. The free-water volume fraction is an additional parameter to the aforementioned parameters of the bi-tensor model.

**Figure 2:**
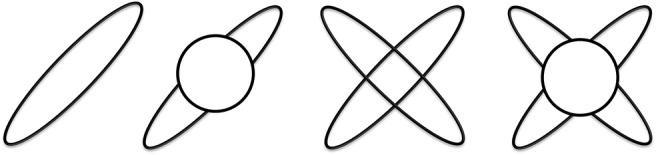
Illustration of the four diffusion models. From left to right: single tensor, single tensor with isotropic compartment, bi-tensor and bi-tensor with an isotropic compartment models.

In all of the models the maximum diffusivity of the tensor compartment is limited by the diffusivity of free water at body temperature. For an illustration of these models see Figure 2. Mathematical details of specific model parametrizations and model fitting procedure are described in the Supplementary Material.

It is important to notice that models with multiple compartments are capable of modeling diffusion signals produced by several fiber populations leading to lower fit errors. However, applying these models to voxels containing a single fiber population may result in noise fitting and thus spurious fiber orientations' and erroneous model parameters.

#### Tract identification

The volume fraction of tissue in a stroke lesion was estimated to be <0.1 by Latour and Warach (2002). Reflecting this decreased parenchymal volume fraction, masks of stroke lesions were created by conservatively thresholding the volume fraction of the isotropic compartment estimated by model (4) at *f*_*iso*_ = 0.9. As such, regions with *f*_*iso*_ larger than this value were excluded from further analysis. The accuracy of the lesion delineation was visually checked by a research fellow (OF). The research fellow could adjust the threshold to obtain a more accurate delineation in case the mask was considered suboptimal.

Separate FA images were derived for each diffusion model and each subject. All FA images were co-registered to the MNI space using an affine registration with 12 degrees of freedom as implemented in FSL v5.0 (Jenkinson et al., 2012). To achieve an accurate registration of the non-lesional brain parts, the masked regions of the chronic stroke subjects were excluded in the registration process. Subsequently, the same transformation was applied to the parameter maps derived from the diffusion models (e.g., the volume fractions). Next, the JHU white-matter tractography atlas, which is also defined in MNI space, was projected onto the data. This tractography atlas contains 20 labeled white matter structures. It was generated by averaging the results of deterministic tractography on 28 normal subjects (mean age 29, M: 17, F: 11) (Mori et al., 2005; Hua et al., 2008). Symmetric WM tracts in this atlas are considered in our analysis. Their functional roles are summarized in Table 1. Additionally, the atlas contains delineations of forceps major and forceps minor.

**Table 1:** White matter tracts of the JHU tractography atlas obtained from deterministic tractography on 28 normal subjects (https://neurovault.org/media/images/264/JHU-ICBM-tracts-maxprob-thr25-1mm.nii.gz, Hua et al., 2008), their approximate location and function.

**Tract name**	**Location**	**Function**
Anterior thalamic radiation (ATR)	Passes from thalamus to pre-frontal cortex	As a part of thalamic radiations, relays sensory and motor data to pre- and post-central cortex
Corticospinal tract (CST)	Connects cerebral motor and somatosensory cortex to medulla and descends into contralateral spinal cord	Facilitates voluntary motor control of the limbs and trunk
Cingulum (all parts): Cingulum 1 -cingulate gyrus (CG); cingulum 2–cingulate hippocampus (CH)	A collection of WM fibers connecting cingulate gyrus (cortex) to the entorhinal cortex	Part of the limbic system of the brain, associated with emotion, visual and spatial skills, working and general memory
Inferior fronto-occipital fasciculus (IFOF)	Connects the occipital lobe with the anterior part of the temporal lobe, running medially and above the optic fibers. It is a direct pathway connecting occipital, posterior temporal orbitofrontal areas	Integration of auditory and visual association cortices with prefrontal cortex. Function is still poorly understood
Inferior longitudinal fasciculus (ILF)	Connects the occipital lobe with the anterior part of the temporal lobe, running above the optic radiation fibers	Integration of auditory and speech nuclei. Function is still poorly understood
Superior longitudinal fasciculus (all parts): SLF and temporal part SLF-T	Major association fiber tract connecting frontal, parietal, and temporal lobes	As a major tract with projections to multiple lobes, it is involved with regulating motor behavior, spatial attention, visual and oculomotor functions, transfer of somatosensory information as well as language
Uncinate fasciculus	A hook-shaped fiber bundle linking anterior parts of the temporal lobe with the lower surface of the frontal lobe	Part of the limbic system which takes part in memory integration

**Figure 3:**
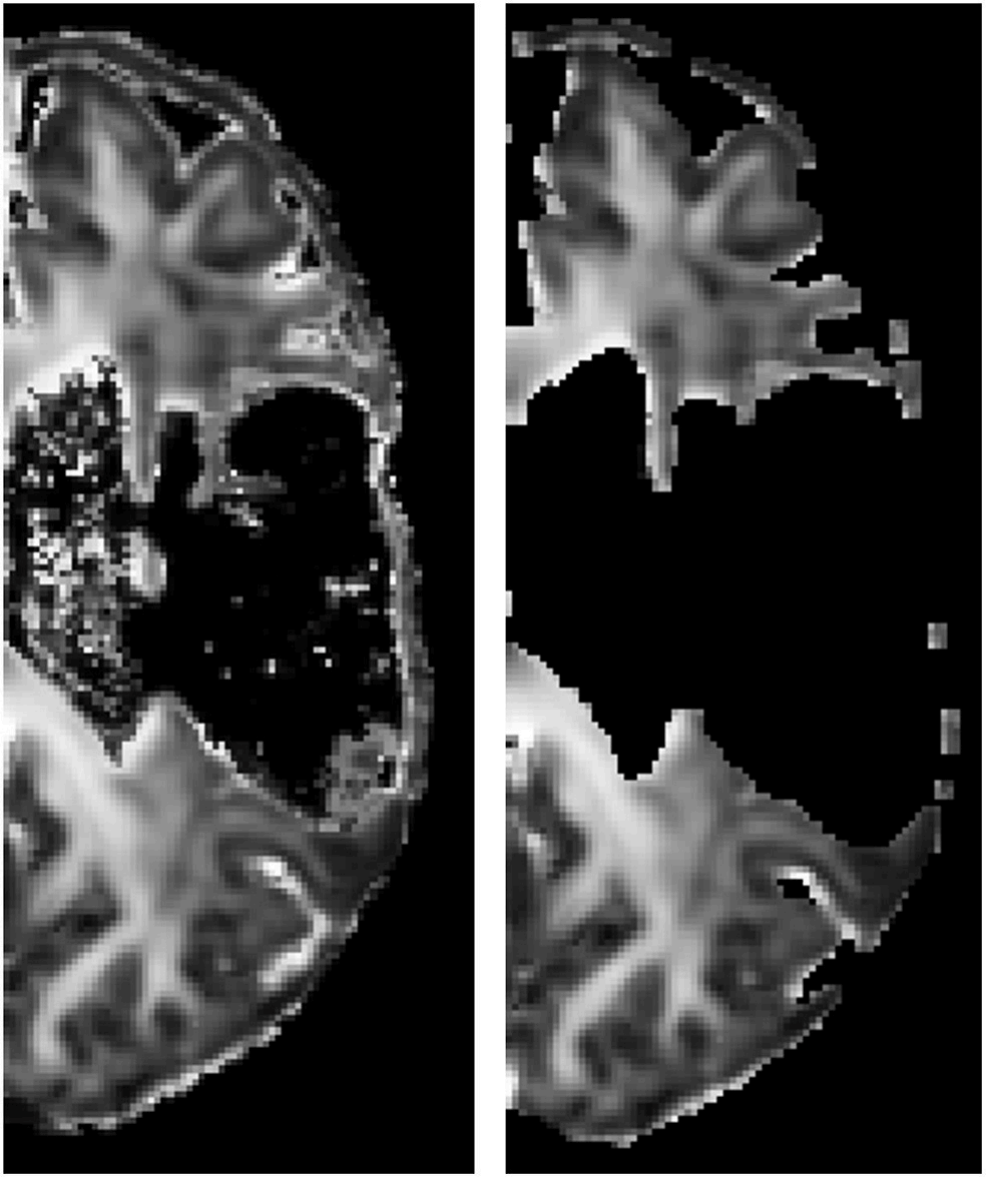
FA image of an affected hemisphere of a patient before (subject space, left) and after (atlas space, right) registration to the atlas space. Lesion mask was applied. The absence of large deformations indicates that the presence of the lesion hardly affected the registration outcome.

Registration to the atlas space is illustrated in Figure 3, showing that no significant warping occurs. Not even for the scans of a patient with a big lesion. For the median and interquartile range of the considered regions of interest computed in the atlas space after masking the lesion out (see Table 2).

**Table 2:** Median and IQR over the study population of the considered regions of interest after masking lesions out, computed in the atlas space.

**Tract name**	**Median (IQR), px**	
	**Right hemisphere**	**Left hemisphere**
Anterior thalamic radiation	11716 (9928.5–12588)	10776 (7265.25–11320.75)
Corticospinal tract	8867 (8451.75–9057.5)	7991.5 (7302.75–8106.75)
Cingulum (all parts)	5021 (4992.75–5053.75)	3781.5 (3721.75–3792.75)
Inferior fronto-occipital fasciculus	11018 (10587.25–11097.5)	12036 (10976–12134,5)
Inferior longitudinal fasciculus	10359.5 (10314–10400)	6964 (6790.5–7004.25)
Superior longitudinal fasciculus (all parts)	14977.5 (14862.5–15017.5)	11881.5 (11186.75–11909.75)
Uncinate fasciculus	2627 (2551.5–2650.75)	1775.5 (1605–1810)

*Names and order of ROIs coincides with Table 1*.

### Data analysis and statistics

The mean parameter values of each model were calculated for every tract and for all subjects. After that, we determined ratios of the mean tract values between contralesional (healthy) and ipsilesional (impaired) hemispheres, which were normalized to the interval between −1 and 1. For example, for FA it was defined as (***FA***_*healthy*_ − ***FA***_*impaired*_)/(***FA***_*healthy*_ + ***FA***_*impaired*_). In this way, WM properties that were balanced between the hemispheres resulted in values close to zero; positive values indicate that the contralesional side has higher FA than the ipsilesional side and vice versa for negative values. In the same manner, the asymmetry was calculated for MD, RD, and AD. For the healthy controls asymmetry was defined between the dominant and non-dominant hemispheres based on the handedness of each subject.

The asymmetries were statistically analyzed using the two-sided Wilcoxon signed rank test. The Benjamini–Hochberg procedure was used to control the false discovery rate at the 5% level (Benjamini and Hochberg, 1995; Benjamini and Yekutieli, 2001), independently for each of the outcome parameters. Comparisons for each model and each diffusivity measure were done independently. WM asymmetries were correlated with the upper limb Fugl-Meyer assessment (FMA) score using Pearson correlation.

## Results

**Figure 4:**
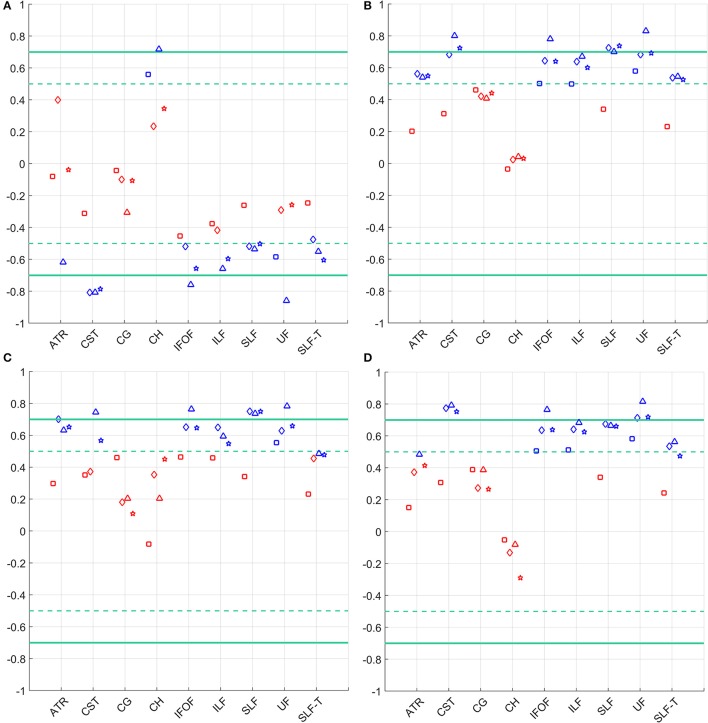
Correlation of the FMA score and asymmetry in **(A)** FA (top left), **(B)** MD (top right), **(C)** AD (bottom left), and **(D)** RD (bottom right) for the considered WM tracts. Statistically significant correlations (*p* < 0.05) are depicted in blue, not significant correlations are shown in red. Models are denoted as follows: single tensor—square, single tensor with isotropic compartment—diamond, bi-tensor—triangle, bi-tensor with isotropic compartment—star. Horizontal dashed lines mark conditional boundaries of moderate (negative) linear relation (correlation of ±0.5), solid lines—strong relation (correlation of ±0.7).

Pearson correlations between FA, MD, AD and RD asymmetries estimated from the four diffusion models with Fugl-Meyer scores are presented in Figure 4 and in the Supplementary Material.

**Figure 5:**
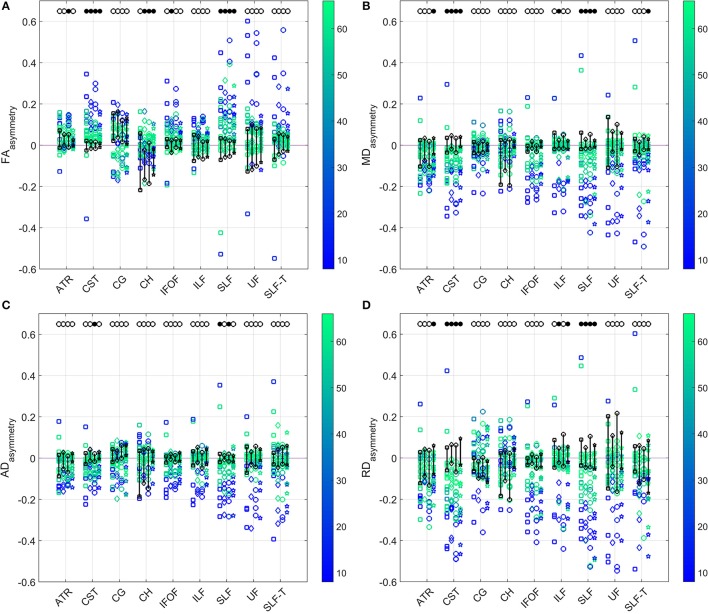
Comparison of the **(A)** FA (top left), **(B)** MD (top right), **(C)** AD (bottom left), and **(D)** RD (bottom right) asymmetries in the WM tracts between patients and controls (range indicated in black) estimated by the four diffusion models. Results for patients are color-coded with their FMA score. Models from left to right for each tract: single tensor (square), single tensor with isotropic compartment (diamond), bi-tensor (triangle), bi-tensor with isotropic compartment (star). Filled bullets at the top of each data series mark the tracts and models for which, after Benjamini–Hochberg correction, the asymmetry of patients is significantly different from the one of controls.

The comparison of asymmetries in diffusion measures between patient and control groups for all four diffusion models is presented in Figure 5. After applying the Benjamini–Hochberg false discovery rate control procedure, the comparisons were deemed to be statistically significant at the 95% confidence level when *p* ≤ 0.016 for FA_asymmetry_, *p* ≤ 0.014 for MD_asymmetry_, *p* ≤ 0.003 for AD_asymmetry_, and *p* ≤ 0.012 for RD_asymmetry_. Patients showed a significant difference from controls in CST asymmetries of FA, MD, and RD for all models, but of AD only for model (3). Similarly, a difference between groups was found in SLF asymmetries of FA, MD, and RD for all models, and of AD only for models (1) and (3). AD_asymmetry_ did not identify other WM tracts as being different between the patient and control groups. None of the models identified statistically significant difference in the cingulum-1, inferior fronto-occipital fasciculus and uncinate fasciculus. For the inferior longitudinal fasciculus, only RD_asymmetry_ for models (2) and (4) and MD_asymmetry_ for model (2) differed significantly between patients and controls.

## Discussion

In this study, we evaluated how WM diffusion properties estimated by four diffusion models of increasing complexity relate to the motor outcome of chronic stroke patients. Particularly, the influence of stroke on symmetric white matter structures was assessed based on the interhemispheric asymmetry of mean tract model parameters. We have not investigated the influence of lesion size and lesion location on motor recovery, which was presented in literature before (e.g., Lo et al., 2010), but focused on four diffusion measures: FA, MD, AD, and RD.

### Model comparison

Diffusion MRI has been used to derive characteristics of the corticospinal tract in chronic stroke using the single tensor model. (Archer et al., 2017) aimed to eliminate bias of free water contamination from the FA estimation with an approach by Pasternak et al. (2009) for modeling a tissue and a free water compartment. We add to this previous work by not only investigating the FA_asymmetry_, but other diffusivity measures (MD, AD, and RD) as well. This is supported by our acquisition protocol with two b-values, as it was discussed in Taquet et al. (2015) that estimation of the parameters in the two-compartment model by Pasternak et al. (2009), NODDI (Zhang et al., 2012), CHARMED (Assaf and Basser, 2005), and DIAMOND (Scherrer et al., 2013)^1^ is ineffective for a single b-value data, unless additional regularization or assumptions are enforced on the model fit. Two-compartment models were also previously applied to multi-shell dMRI data by, for example, (Pierpaoli and Jones, 2004; Pasternak et al., 2012; Hoy et al., 2014).

The WM asymmetries were assessed using four diffusion models of increasing complexity: (1) single tensor, (2) single tensor with isotropic compartment, (3) bi-tensor, and (4) bi-tensor with isotropic compartment. The addition of each compartment to the models in this study permits modeling of an additional physical phenomenon (i.e., free water and crossing fibers) and contributes to the interpretation of the results. It is important to notice that the outcome parameters of the selected diffusion models are directly comparable to each other. For example, the frequently used ball-and-stick model (Behrens et al., 2003) yields volume fractions of the fiber populations that cannot be directly compared to FA. However, more complex models do not everywhere in the brain reflect the underlying white matter anatomy. Applying a two tensor model to the diffusion data from a single fiber tract may lead to overfitting and meaningless outcome parameters. This is described in Yang et al. (2016). Therefore, it cannot be taken for granted that more complex models are always performing better, especially in case of pathologies.

Damage to the CST after stroke has been extensively investigated in Stinear et al. (2007); Schaechter et al. (2008, 2009), and Cunningham et al. (2015). A linear correlation was found between the CST integrity and increased activation of the contralesional primary sensorimotor cortex (Schaechter et al., 2008). Qiu et al. (2011) showed a close relationship between the FA asymmetry and clinical outcome measures. The current study expands on this by exploring this relation for multiple WM properties assessed by four diffusion models. We did not find a significant Pearson correlation between the asymmetry of single tensor diffusion measures and the upper-limb FMA in most of the tracts, not even in the CST. The other models do show significant correlations between the CST asymmetry and the FMA score. The highest correlation and statistical significance are for FA and RD CST asymmetries. Except for the cingulum (tracts 3 and 4), MD, AD, and RD asymmetry derived from models (2) to (4) showed significant correlation with the FMA for all other tracts. However, it was less consistent among tracts for the FA asymmetry. This may indicate that not only CST integrity, but also the state of other white matter tracts could be indicative of the patient's motor abilities after stroke. A similar conclusion was previously reached based on a voxel-wise FA analysis (Schaechter et al., 2009).

Our findings indicate that accounting for the free water by adding an isotropic compartment to the single tensor model already significantly increases the sensitivity of diffusion outcomes. Differences in the outcomes of different models may suggest that for specific tracts and especially in the areas hampered by partial volume effects, it can be beneficial to go beyond the single tensor model when relating the results to the patient motor function.

### Affected white matter tracts

We investigated the asymmetry in FA, MD, AD, and RD and tested whether the asymmetry was significantly different from the same measures for the control group and whether it correlated with the FMA score of the patients. As a higher FA may be related to a better quality of WM tracts (Hüppi and Dubois, 2006; Teipel et al., 2010; Winston, 2012), statistically significant correlations of FA_asymmetry_ with the motor score are negative. The rest of the diffusion properties have an inverse relation, e.g., lower MD indicates higher membrane density, leading to significant correlations of MD, AD, and RD asymmetries with FMA being negative. Moreover, prior research predominantly focused on the CST in general and the posterior limb of the internal capsule in particular, because these structures are regularly associated with stroke damage (Jang, 2011; Park et al., 2013; Song et al., 2015). Instead, we chose to pursue a more comprehensive analysis involving diffusivity parameters in nine symmetric tracts as listed above.

As might be expected, the WM tracts asymmetry in healthy controls fluctuated around zero. After false discovery rate correction, FA, MD, and RD, but not AD, asymmetries differed between patients and controls in CST and SLF. Model (1) also failed to identify the FA_asymmetry_ in SLF. Asymmetry in SLF has not been reported previously. However, the dorsal component of SLF originates in the superior and medial parietal cortex and terminates in the dorsal and medial cortex of the frontal lobe and in the supplementary motor cortex. Therefore, changes in the SLF with respect to the control group could have been expected as all patients in this study suffered from a certain extend of motor impairment. Overall, patients with more severe impairment, indicated by lower FMA scores, displayed indeed higher asymmetry of their WM properties.

### Limitations

Diffusion properties of the cingulum (tracts 3 and 4) did not correlate with FMA and showed higher asymmetries even for the control group (Figures 4, 5). Information about the dominant and the impaired hand of the patients may suggest that handedness of subjects could have influenced the sign of the WM asymmetry. A number of studies investigated the effects of handedness on the human brain. Differences in volumes of gray and white matter areas were detected by Herve et al. (2006). A voxel-based statistical analysis found higher FA in the left arcuate fasciculus in consistent right-handers (Büchel et al., 2004). However, this was not confirmed in a similar study by Park et al. (2004). Despite the fact that right hand preference might be expected to result from asymmetries in the motor cortex, it is stronger correlated with asymmetries in language-processing structures (Toga and Thompson, 2003). A more recent study by Powell et al. (2012) suggests a greater effect of sex than handedness on FA asymmetry. McKay et al. (2017) found significantly higher FA-values for left-handed individuals in all major lobes and in the corpus callosum compared to the right-handed subjects. As such, results regarding handedness effects on WM have not been entirely consistent across different studies.

In our work we rely on an assumption that stroke causes a much larger asymmetry than the handedness of a person. We did compare the effect of stroke to the baseline asymmetries in diffusion properties of our control group, which are quite symmetrically distributed around zero (except for the cingulum as mentioned above) and are in range that is at least twice smaller than that of the patient group. Although we are aware that this approach has drawbacks, it is frequently performed to avoid oversegmentation of the cohort, i.e., dividing it into groups based on handedness, (Luft et al., 2004; De Vico Fallani et al., 2013). Practically, it enabled us to include a sufficiently large subject group to achieve statistically valid results and conclusions. Such an approach is similar to the underlying assumption of many other stroke studies (Bhagat et al., 2008; Lindenberg et al., 2010; Puig et al., 2010; Park et al., 2013; Cunningham et al., 2015; Archer et al., 2017).

As a limitation we would also like to mention the absence of a ground truth, as the true diffusion properties of WM in stroke patients are unknown. This limits our ability to evaluate the performance of the diffusion models, but this is a common problem of *in-vivo* studies into brain structures, allowing only indirect assessments. Another factor is the smaller size of the control group compared to the patient group. We assume that the variation in the outcome parameters (e.g., FA_asymmetry_, MD_asymmetry_, etc.) of the controls is much smaller than those of the patients. Accordingly, the smaller group size of the controls should not hinder the comparison. This was confirmed in our power analysis, which indicated that the chance of detecting a large effect is 90%. That is why we consider our study design to be sufficient to fulfill its aims. However, a design with equal group size would have further enhanced the statistical power of the study.

### Outlook

Differences in the parameter outcomes of different models may suggest that for specific tracts and especially in brain areas contaminated by partial volume effects, it can be beneficial to go beyond the single tensor model when relating the outcomes to the patients' motor function. Approaching stroke dMRI equipped with more sophisticated models could be helpful in studying scans at a more acute phase, when it is important to make a prediction of recovery and adjust therapy. This could be most beneficial for so-called non-fitters to the 70% recovery rule (see, for example, Winters et al., 2015) and differentiating between necrotic and still salvageable tissue. Our results suggest that employing multi-compartment diffusion models should be tested to investigate WM changes in stroke patients longitudinally, starting from the acute phase.

## Conclusions

- FA and RD asymmetries are an indication of white matter alterations after stroke and related with the patients' motor outcome.
- As for selecting a diffusion model, the bi-tensor model with isotropic compartment should be used if permitted by the dMRI acquisition protocol (i.e., sufficient diffusion directions and multiple b-values). Otherwise, a single tensor model with isotropic compartment is a good alternative.
- Not only the cortical-spinal tract, but also the superior longitudinal fasciculus integrity values are significantly affected by stroke as indicated by the group comparison between patients and controls.
- Approaching dMRI in stroke patients with sophisticated diffusion models instead of a single tensor model leads to a higher sensitivity and should be tested in scans at a more acute phase, when it is important to make a prediction of recovery and adjust therapy. This could be most beneficial for so-called non-fitters to the 70% recovery rule (see, for example, Winters et al., 2015) and for differentiating between necrotic and still salvageable tissue.
- Employing multi-compartment diffusion models should be tested to detect WM changes in stroke patients longitudinally, starting from the acute phase.

## Ethics approval and consent to participate

All subjects gave written informed consent in accordance with the Declaration of Helsinki. The protocol was approved by the Medical Ethics Reviewing Committee of the VU Medical Center, Amsterdam (protocol number 2014.140, Dutch Central Committee on Research Involving Human Subjects, CCMO, protocol number NL47079.029.14).

## Author contributions

Conception of the study was conducted by GK and FH. All authors participated in design of the study. GK supervised the data acquisition. OF analyzed and interpreted the data with input and support from LvV, AS, FvdH, and FV. OF and FV drafted the manuscript. All authors revised the manuscript critically for important intellectual content. All authors read and approved the manuscript.

### Conflict of interest statement

The authors declare that the research was conducted in the absence of any commercial or financial relationships that could be construed as a potential conflict of interest.

## Footnotes

^1^NODDI, Neurite Orientation Dispersion and Density Imaging; CHARMED, Composite hindered and restricted model of diffusion; DIAMOND, DIstribution of 3-D Anisotropic MicrOstructural eNvironments in Diffusion-compartment imaging.
