# Supplementary material for: Comparison of Multi-Tensor Diffusion Models' Performance for White Matter Integrity Estimation in Chronic Stroke
Source: Front Neurosci. 2018 Apr 23;12:247. doi: 10.3389/fnins.2018.00247 (PMC5925961; doi:10.3389/fnins.2018.00247)
Supplement: Supplementary file 1 [file DataSheet1.DOCX]

Supplementary Material

Comparison of Multi-Tensor Diffusion Models’ Performance for White Matter Integrity Estimation in Chronic Stroke

O.G. Filatova^1,2^, L.J. van Vliet^2^, A.C. Schouten^1,3^, G.Kwakkel^4^, F.C.T. van der Helm^1^ and F.M. Vos^2,5*^, on behalf of the 4D EEG consortium

*** Correspondence:** F.M. Vos: f.m.vos@tudelft.nl

# Diffusion modeling

Assuming a mono-exponential and Gaussian diffusion along the principal directions (given by the eigenvectors) of the separate fiber compartments, the measured dMR signal $S_{j}$ can be modeled as a sum of up to two fiber bundles and an isotropic compartment in the following way:

$$S_{j}=S_{0}\left(\sum_{i=1,2} f_{i}\exp\left(-\mathbf{b}_{j}\mathbf{g}_{j}^{T}\mathbf{D}_{i}\mathbf{g}_{j}\right)+f_{iso}\exp\left(-\mathbf{b}_{j}D_{iso}\right)\right)$$

where $S_{0}$ is the signal without diffusion weighting, $f_{i}$ and $f_{\mathrm{iso}}$ are the volume fractions of different compartments such that $\sum_{i=1,2} f_{i}+f_{iso}=1$, $\mathbf{g}_{j}$ is a gradient direction, $\mathbf{b}_{j}$ is the diffusion weighting coefficient, $\mathbf{D}_{i}$ is the diffusion tensor of the $i^{\mathrm{th}}$ fiber, and $D_{\mathrm{iso}}$ is the scalar coefficient of isotropic diffusion. Here directions of diffusion sensitizing gradients and the amount of weighting are inputs, depending on the acquisition protocol. Different models are obtained by adjusting the number of fibers, constraints on diffusion tensors and presence or absence of the isotropic diffusion process. The assumption of the mono-exponential signal decay is valid up to at least 2000 s/mm^2^ (Yoshiura et al., 2001), or even up to 3000 s/mm^2^ as reported in the multiple sclerosis study by (Assaf et al., 2002).

To estimate the unknowns, the diffusion tensor can be parametrized in polar coordinates. Parameters of the considered models are listed below.

Single tensor: .

Single tensor with an isotropic compartment: .

Bi-tensor model: .

Bi-tensor with an isotropic compartment: .

Here $\lambda_{1},\lambda_{2},\lambda_{3}$ are eigenvalues of the diffusion tensor, $\lambda_{||},\lambda_{\perp}$ denote the axial and perpendicular diffusion respectively, $\psi_{i}$, $\theta_{i}$ and determine the $i^{\mathrm{th}}$ fiber orientation in polar coordinates.

The parameter vector is obtained by maximizing the log likelihood of the joint probability density function for diffusion-weighted signals with independent noise realizations. It is done using our constrained non-linear optimization routine in Matlab (The MathWorks, Natick, MA). The optimization problem can be formulated as follows.

Let $\widetilde{\text{S}}_{j,\sigma}$ be the measured diffusion weighted image (DWI) with diffusion weighting $\mathbf{b}_{j}$ in direction $\mathbf{g}_{j}$. It is affected by Rician noise with standard deviation $\sigma$(Gudbjartsson and Patz, 1995). Therefore, for the parameter vector $\mathbf{\theta}$, the probability density function (PDF) of the measured signal is given by

$p(\widetilde{\text{S}}_{j,\sigma}|\mathbf{\theta})=\frac{\widetilde{\text{S}}_{j,\sigma}}{\sigma^{2}}\exp\left(\frac{\widetilde{\text{S}}_{j,\sigma}^{2}+S_{\mathbf{\theta},j}^{2}}{2\sigma^{2}}\right)I_{0}\left(\frac{\widetilde{\text{S}}_{j,\sigma}S_{\mathbf{\theta},j}}{\sigma^{2}}\right)$,

where $I_{0}(\cdot)$ is the zeroth order modified Bessel function of the first kind, . Due to statistical independence of the DWIs, the joint probability density function $p(\widetilde{\mathbf{S}}_{\sigma}|\mathbf{\theta})$ of the signal profile $\widetilde{\mathbf{S}}_{\sigma}$ is equal to the product of the marginal distributions for the measured signals $\widetilde{\text{S}}_{j,\sigma}$ in each of the $N_{g}$ diffusion weighted directions $\mathbf{g}_{j}$:

$$p\left(\widetilde{\mathbf{S}}_{\sigma}|\mathbf{\theta}\right)=\prod_{j=1}^{N_{g}} p\left(\widetilde{\text{S}}_{j,\sigma}|\mathbf{\theta}\right)$$

The parameter values can be estimated by maximizing the log likelihood function of $\mathbf{\theta}$ given $\widetilde{\mathbf{S}}_{\sigma}$ (Sijbers et al., 2004):

$\widehat{\mathbf{\theta}}_{\text{MLE}}=\mathop{\mathrm{argmax}}\limits_{\mathbf{\theta}} \left\{\ln\left(p(\widetilde{\mathbf{S}}_{\sigma}|\mathbf{\theta})\right)\right\}$.

Maximum likelihood estimation (MLE) has a number of beneficial properties for diffusion estimation in crossing fiber bundles (Caan et al., 2010). First of all, under very general conditions, MLE asymptotically reaches the Cramér-Rao lower bound (CRLB). This is a theoretical lower bound on the variance of any unbiased estimator. Secondly, the MLE is consistent, which means that it asymptotically ($N_{g}\to\infty$) converges to the true value of the parameter in a statistically well-defined way (Van den Bos, 2007). Optimization is performed using Levenberg–Marquardt algorithm.

**References**

Assaf, Y., Ben-Bashat, D., Chapman, J., Peled, S., Biton, I.E., Kafri, M., et al. (2002). High b-value q-space analyzed diffusion-weighted MRI: Application to multiple sclerosis. *Magnetic Resonance in Medicine* 47(1)**,** 115-126. doi: 10.1002/mrm.10040.

Caan, M.W., Khedoe, H., Poot, D., Den Dekker, A., Olabarriaga, S., Grimbergen, K., et al. (2010). Estimation of diffusion properties in crossing fiber bundles. *IEEE Trans Med Imaging* 29(8)**,** 1504-1515.

Gudbjartsson, H., and Patz, S. (1995). The Rician Distribution of Noisy MRI Data. *Magnetic resonance in medicine : official journal of the Society of Magnetic Resonance in Medicine / Society of Magnetic Resonance in Medicine* 34(6)**,** 910-914.

Sijbers, J., Den Dekker, A.J., Scheunders, P., and Van Dyck, D. (2004). Maximum likelihood estimation of signal amplitude and noise variance from MR data. *Magn Reson Med* 51(3)**,** 586-594.

Van den Bos, A. (2007). *Parameter Estimation for Scientific and Engineers.* Hoboken, NJ: Wiley.

Yoshiura, T., Wu, O., Zaheer, A., Reese, T.G., and Sorensen, A.G. (2001). Highly diffusion-sensitized MRI of brain: Dissociation of gray and white matter. *Magnetic Resonance in Medicine* 45(5)**,** 734-740. doi: 10.1002/mrm.1100.
