# Supplementary material for: Comparison of Multi-Tensor Diffusion Models' Performance for White Matter Integrity Estimation in Chronic Stroke
Source: Front Neurosci. 2018 Apr 23;12:247. doi: 10.3389/fnins.2018.00247 (PMC5925961; doi:10.3389/fnins.2018.00247)
Supplement: Supplementary file 2 [file Table3.DOCX]

Supplementary Material

Comparison of Multi-Tensor Diffusion Models’ Performance for White Matter Integrity Estimation in Chronic Stroke

O.G. Filatova^1,2^, L.J. van Vliet^2^, A.C. Schouten^1,3^, G.Kwakkel^4^, F.C.T. van der Helm^1^ and F.M. Vos^2,5*^, on behalf of the 4D EEG consortium

*** Correspondence:** F.M. Vos: f.m.vos@tudelft.nl

# Additional results

Table 3. Correlation coefficients of the white matter properties of the considered tracts with the Fugl-Meyer upper extremity assessment score for each diffusion model and their corresponding p-values. Significant correlations are marked in bold.

|  | | | ATR | CST | CG | CH | IFOF | ILF | SLF | UF | SLF-T |
| --- | --- | --- | --- | --- | --- | --- | --- | --- | --- | --- | --- |
| FA | ST | R | -0.08 | -0.3 | -0.043 | **0.56** | -0.45 | -0.38 | -0.26 | **-0.58** | -0.25 |
|  |  | p | 0.75 | 0.21 | 0.87 | **0.016** | 0.059 | 0.12 | 0.3 | **0.011** | 0.32 |
|  | ST-Iso | R | 0.4 | **-0.81** | -0.099 | 0.23 | **-0.51** | -0.42 | **-0.52** | -0.29 | **-0.48** |
|  |  | p | 0.1 | **5E-5** | 0.69 | 0.35 | **0.027** | 0.09 | **0.028** | 0.24 | **0.046** |
|  | BT | R | **-0.62** | **-0.81** | -0.31 | **0.72** | **-0.76** | **-0.66** | **-0.54** | **-0.86** | **-0.55** |
|  |  | p | **6E-3** | **5.3E-5** | 0.22 | **8E-4** | **2.6E-4** | **3E-3** | **0.02** | **5.2E-6** | **0.018** |
|  | BT-Iso | R | -0.04 | **-0.79** | -0.11 | 0.35 | **-0.65** | **-0.6** | **-0.5** | -0.26 | **-0.61** |
|  |  | p | 0.88 | **1E-4** | 0.68 | 0.16 | **3.1E-3** | **9E-3** | **0.034** | 0.3 | **7.8E-3** |
| MD | ST | R | 0.2 | 0.31 | 0.46 | -0.035 | **0.5** | **0.5** | 0.34 | **0.58** | 0.23 |
|  |  | p | 0.42 | 0.21 | 0.05 | 0.9 | **0.034** | **0.035** | 0.17 | **0.012** | 0.36 |
|  | ST-Iso | R | **0.56** | **0.68** | 0.42 | 0.024 | **0.65** | **0.64** | **0.73** | **0.68** | **0.54** |
|  |  | p | **0.015** | **1.7E-3** | 0.08 | 0.92 | **3.9E-3** | **4.3E-3** | **6.6E-4** | **1.8E-3** | **0.02** |
|  | BT | R | **0.54** | **0.8** | 0.41 | 0.04 | **0.78** | **0.67** | **0.7** | **0.83** | **0.55** |
|  |  | p | **0.02** | **6.3E-5** | 0.09 | 0.87 | **1.3E-4** | **2.3E-3** | **1.2E-3** | **1.9E-5** | **0.019** |
|  | BT-Iso | R | **0.55** | **0.72** | 0.44 | 0.03 | **0.64** | **0.6** | **0.74** | **0.69** | **0.53** |
|  |  | p | **0.018** | **6.8E-4** | 0.067 | 0.9 | **4.2E-3** | **8E-3** | **4.7E-4** | **1.4E-3** | **0.025** |
| AD | ST | R | 0.3 | 0.35 | 0.46 | -0.08 | 0.46 | 0.46 | 0.34 | **0.55** | 0.23 |
|  |  | p | 0.23 | 0.15 | 0.054 | 0.75 | 0.052 | 0.055 | 0.17 | **0.017** | 0.35 |
|  | ST-Iso | R | **0.7** | 0.37 | 0.18 | 0.35 | **0.65** | **0.65** | **0.75** | **0.63** | 0.46 |
|  |  | p | **1E-3** | 0.12 | 0.47 | 0.15 | **3.4E-3** | **3.5E-3** | **3.2E-4** | **5E-3** | 0.058 |
|  | BT | R | **0.63** | **0.74** | 0.2 | 0.2 | **0.76** | **0.59** | **0.74** | **0.78** | **0.49** |
|  |  | p | **5E-3** | **3.9E-4** | 0.41 | 0.42 | **2.2E-4** | **9.3E-3** | **4.9E-4** | **1.2E-4** | **0.04** |
|  | BT-Iso | R | **0.65** | **0.57** | 0.11 | 0.45 | **0.65** | **0.55** | **0.75** | **0.66** | **0.48** |
|  |  | p | **3.3E-3** | **0.014** | 0.67 | 0.06 | **3.7E-3** | **0.019** | **3.4E-4** | **2.9E-3** | **0.045** |
| RD | ST | R | 0.15 | 0.31 | 0.39 | -0.051 | **0.51** | **0.51** | 0.34 | **0.58** | 0.24 |
|  |  | p | 0.55 | 0.21 | 0.11 | 0.84 | **0.032** | **0.03** | 0.17 | **0.011** | 0.33 |
|  | ST-Iso | R | 0.37 | **0.77** | 0.27 | -0.13 | **0.64** | **0.64** | **0.68** | **0.71** | **0.54** |
|  |  | p | 0.13 | **1.6E-4** | 0.27 | 0.6 | **4.5E-3** | **4.1E-3** | **2.1E-3** | **8.9E-4** | **0.022** |
|  | BT | R | **0.48** | **0.79** | 0.39 | -0.08 | **0.76** | **0.68** | **0.67** | **0.82** | **0.56** |
|  |  | p | **0.04** | **9E-5** | 0.11 | 0.75 | **2.2E-4** | **1.8E-3** | **2.6E-3** | **3.6E-5** | **0.015** |
|  | BT-Iso | R | 0.41 | **0.75** | 0.27 | -0.29 | **0.64** | **0.63** | **0.66** | **0.72** | **0.47** |
|  |  | p | 0.09 | **3.1E-4** | 0.29 | 0.24 | **4.3E-3** | **5.5E-3** | **2.8E-3** | **7.7E-4** | **0.047** |
